# Consumer-Relevant Endpoints in Nutritional Supplement Research: A Case of Multivitamin and Mineral Supplements

**DOI:** 10.1016/j.cdnut.2026.107708

**Published:** 2026-05-02

**Authors:** Rocco Sheldon, Ee Moon Chin, Andrew Gan, Morya Wadodkar, Elena Sandalova, Muhammad Daniel Azlan Mahadzir

**Affiliations:** 1 College of Medicine and Health, University of Birmingham, Birmingham, United Kingdom; 2 Department of Chemistry, National University of Singapore, Singapore, Singapore; 3 Bristol Medical School, University of Bristol, Bristol, United Kingdom; 4 EVIDENCEPOINTSA, Singapore, Singapore; 5 SingHealth Duke-NUS Global Health Institute, Duke-NUS Medical School, Singapore, Singapore; 6 SingHealth Office for Sustainable Health, Singapore, Singapore

**Keywords:** multivitamin supplements, multimineral supplements, dietary supplementation, quality of life, well-being, public health

## Abstract

Multivitamin and mineral (MVM) supplements occupy a unique place in modern healthcare. They are one of only a few interventions the public actively embraces, despite clear guidelines that do not recommend routine MVM use for healthy people, because a balanced diet should provide all essential nutrients. This public adoption of MVMs creates a paradox when people say they take MVMs to support their well-being, yet scientific studies do not capture or measure what that actually entails. It also presents an opportunity to explore why this divergence exists, and in doing so, explore how people understand health and well-being. This review argues that trials of MVM supplementation in the general adult population are methodologically misaligned with the outcome consumers value, relying on endpoints and instruments that are neither representative nor sufficiently sensitive. Beyond any placebo effect, the summative effect of addressing multiple subclinical micronutrient deficiencies represents a potential, plausible biological basis for improved quality of life (QoL). Future MVM studies should be designed to comprehensively evaluate QoL and related outcomes (e.g., cognition, fatigue, and resilience) to align with reasons why adults take MVM supplements.

## Introduction

As medical doctors and clinical researchers, we inhabit a world of evidence-based practice. We are guided by large-scale, randomized, double-blind, placebo-controlled trials [randomized controlled trial (RCTs)] as the reference standard of our profession. Regarding the topic of multivitamin and mineral (MVM) supplements for the general adult population, the trials have spoken quite clearly.

The Physicians’ Health Study II (PHS-II) [1], a large RCT enrolling 14,641 male physicians, found that long-term daily MVM use had no significant effect on major cardiovascular events or all-cause mortality. Although it did find a modest reduction in total cancer incidence, it showed no effect on cancer mortality. The COcoa Supplement and Multivitamin Outcomes Study (COSMOS) [2], which randomly assigned >21,442 older males and females, also found that MVM supplementation did not reduce the risk of cardiovascular disease (CVD) or cancer. Similar results have been reported in large cohort studies, with the largest finding no association between MVM use and mortality benefits across 390,124 United States adults [3].

Meta-analyses have confirmed these findings, concluding that for healthy adults without specific nutritional deficiencies, MVMs do not reduce the risk of death from any cause [4,5]. This evidence has led to clear public health recommendations. Authoritative bodies consistently advise against the routine use of MVMs for disease prevention [6], suggesting that a healthy lifestyle and balanced diet are far more effective strategies.

And yet, the public has, by and large, taken this advice lightly. MVMs are the most common dietary supplement, taken by more than one-third of all United States adults [7,8]. Eighty-eight percent of adults in Europe have taken a dietary supplement at some point in their life, according to the IPSOS 2022 survey of 14 European Union countries [9]. This creates a significant paradox: continued investment in a practice that is not supported by scientific evidence. What needs to be addressed is whether this paradox is due to consumers being misinformed and/or if the scientific community has failed to measure consumer-relevant endpoints. In existing studies, such endpoints and perceived health have been measured using quality of life (QoL), which, therefore, forms the focus of the review.

## Methods

This review aimed to explore the evaluation of QoL indicators following MVM supplementation among adults. The authors posit that despite mixed clinical outcomes in all registered trials, there is a possible impact on QoL that leads to high rates of MVM consumption in adults. A targeted literature search was conducted to identify studies informing this perspective on QoL outcomes in MVM research. The search was used to characterize the types of studies and consumer-relevant outcomes reported, and to support the interpretive discussion below. Four databases (PubMed, EMBASE, Web of Science, and Cochrane CENTRAL) were searched from inception to 1 November, 2025. The search used Medical Subject Headings and terms related to “MVM,” “multivitamin,” “mineral supplementation,” “quality of life,” “HRQoL,” and the names of QoL tools using Boolean operators. The search strategy for PubMed is outlined in Appendix A.

The authors gathered information on outcome evaluations used in these studies, which are presented as a narrative synthesis to answer the main exploratory question of this review.

## Results and Discussion

### The “belief gap”: measuring health compared with feeling healthy

The answer may lie in a fundamental disconnect in perceived outcomes. Clinicians and healthcare professionals are trained to measure morbidity and mortality. The public, however, is often pursuing well-being and vitality [10]. Evidently, the two are not the same.

This “belief gap” was clearly illustrated in a large cross-sectional analysis of the 2012 National Health Interview Survey, which included >21,603 United States adults [11]. The study compared MVM users and nonusers across a range of outcomes. When it came to “clinically measurable health outcomes,” such as a history of 10 chronic diseases, the presence of 19 current health conditions, or scores on the Kessler 6-Item Psychological Distress Scale, the study found no differences between the two groups. This aligns perfectly with the other RCTs data.

However, the study also asked participants for a subjective assessment. On this measure, MVM users self-reported 30% better overall health than nonusers [11].

This single finding is, perhaps, the most important piece of the puzzle. MVM users feel healthier, even in the absence of objective, clinically measurable differences in their disease burden. This suggests the consumer’s goal is not necessarily the prevention of a future, abstract clinical event like a myocardial infarction, but the preservation of their current health and the enhancement of their subjective QoL.

If this is true, then large-scale RCTs, while methodologically sound, may fail to address this question. The public is asking, “Will this help me feel better?” The scientific community has been structured to answer, “Will this prevent disease or death?”

### A methodological misalignment: explaining persistent null results in scientific studies

The null findings from MVM trials may be less of a definitive verdict on supplements and more of an indictment of our methodological approach. The design of these trials is often geared toward a pharmaceutical model, a single agent intended to produce a large effect on a specific “hard” clinical endpoint [3]. This approach is ill-suited for a low-dose, nonspecific nutritional intervention in a general population.

This mismatch manifests in 3 critical ways:

#### Afterthought endpoints

The PHS-II and COSMOS trials were designed, funded, and powered to detect changes in cancer, CVD, and mortality. These are morbidity and mortality outcomes. QoL, if measured at all, is almost universally relegated to a secondary or tertiary outcome, often with insufficient thought given to its measurement. The intervention (a subtle nutritional supplement) is simply not aligned with the massive, binary outcome (e.g., all-cause mortality) it is being tested against.

#### Inappropriate study population

Our largest trials, including PHS-II, enrolled a population of “healthy” male physicians, who are likely to be well-nourished at baseline. As the study’s own authors noted in their limitations, such a population may be “too well-nourished to benefit from multivitamin supplementation" [12]. A nutritional intervention designed to correct a deficiency will, by definition, have no effect in a population that is not deficient.

#### Inappropriate QoL measurement tool

When trials attempt to measure QoL, they systematically select the wrong instruments for the population and the intervention. This is not a minor issue; it is a foundational conceptual and psychometric failure that makes a null finding almost inevitable. This failure has 2 parts:

• The conceptual conflation: Clinical research often conflates “QoL” with “health-related QoL” (HRQoL) [13]. True QoL is a broad, holistic, and subjective concept, encompassing an individual’s “perception of their position in life” across multiple domains, including social, spiritual, and personal autonomy [14]. In contrast, HRQoL is a much narrower construct, intended to measure only the “patient’s general perception of the effect of illness and treatment on physical, psychological, and social aspects of life” [15]. As Karimi and Brazier [16] have argued, many HRQoL questionnaires are, in fact, just measures of “self-perceived health status.”
• The psychometric failure: This conceptual error leads researchers to select generic HRQoL instruments, most commonly the 36-Item Short-Form Health Survey (SF-36) [17] and the EuroQol 5-dimension (EQ-5D) [18]. These instruments are designed to measure functional deficits and the impact of disease*.* When researchers administer these “health status” surveys to a healthy population, they create a predictable ceiling effect [19]. The healthy participant scores at or near the maximum score at baseline. As one analysis of an antioxidant supplementation trial noted, participants in both the placebo and supplemented groups “used a maximum score (100) at baseline, making it impossible to detect any improvement” [20]. Furthermore, instruments like the EQ-5D have been shown to have poor-to-moderate responsiveness, meaning they are insensitive to the very subtle changes in well-being that a nutritional supplement might plausibly induce.

In essence, the scientific approach has been to give a subtle supplement to a healthy population and then measure the effect with an instrument that is psychometrically incapable of detecting subtle, positive changes in well-being. The resulting “null finding” is a methodological artifact.

### Measuring the preservation of QoL

If we are to bridge the gap between scientific evidence and public behavior, we must begin by measuring what matters to the public. Two surveys of a combined 22,156 United States adults both revealed the most prevalent reasons for taking MVM supplements as improving and maintaining overall health and wellness [21,22]. Therefore, this means shifting our focus from the prevention of disease to the preservation of health and subjective QoL.

The ancillary studies to the COSMOS trial provide a perfect template. Although the main trial, looking for CVD and cancer endpoints, was negative, the COSMOS-Web [23] and COSMOS-Mind [24] substudies looked at cognition. Critically, they did not use a generic tool; they used a specific and sensitive battery of neuropsychological tests, including the Modified Rey Auditory Verbal Learning Test, immediate recall test [25]. With this precise instrument, they did find a statistically significant benefit: daily MVM supplementation improved global cognition and executive function in older adults. Although the COSMOS-Mind secondary analysis found no reductions in the incidence of mild cognitive impairment or dementia, the study was underpowered for these relatively infrequent outcomes over a 3-y follow-up, and the MVM arm still showed favorable cognitive trajectories at the point patients converted to mild cognitive impairment [26]. This is the key. When a specific, sensitive tool is used to measure a specific, plausible outcome, it can reveal effects that might otherwise remain undetected with a generic tool or disease incidence.

We must apply this same logic to QoL. Future trials should replace the use of generic, function-based instruments and utilize instruments specifically designed to measure subjective, psychosocial QoL in general populations, independent of physical health status. QoL in healthy adults relates less to the absence of limitations as in diseased populations, but rather to the presence of positive domains, their cognition, positive mood, perceived energy, sleep quality, and resilience to stress. These components align more closely to the lived experience of healthy adults, rather than disease-related limitations in physical, social, and role activities as in HRQoL tools such as SF-36 [17].

Numerous tools have been validated for measuring QoL in healthy adults. The 5-item WHO Well-Being Index (WHO-5) is a short and widely used questionnaire to assess psychological well-being, shown to be applicable across research fields in all countries, with high specificity and sensitivity at detecting depression [27,28]. Another validated tool designed for the general adult population is the ICEpop CAPability measure for Adults (ICECAP-A). Recommended by the United Kingdom’s National Institute of Health and Care Excellence to measure the impact of social care interventions, this tool covers 5 domains developed by qualitative United Kingdom–based research: enjoyment, achievement, stability, attachment, and control [29]. The Psychological General Well-Being Index (PGWBI) is a 22-item questionnaire that measures general and psychological well-being across 5 domains: general health, positive well-being, anxiety, depression, vitality, and self-control. Both the full and shorter 6-item instruments have been validated in general populations and can effectively discriminate self-perceived health [30]. Instruments to assess specific domains of QoL, such as fatigue (e.g., Chalder Fatigue Scale), vitality (e.g., Subjective Vitality Scale), and perceived immune fitness, have also been validated in general populations [[31], [32], [33]].

Moving along the age scale, the Control, Autonomy, Self-realisation, Pleasure is a 19-item measure developed specifically to capture positive well-being in older age [34]. Its entire theoretical framework is designed to be independent of health and financial circumstances, making it the perfect tool to test the MVM hypothesis [35]. It is already the reference standard for QoL measurement in major longitudinal studies, such as the English Longitudinal Study of Ageing [36] and the Survey of Health, Ageing and Retirement in Europe, which used the shorter 12-item version [37]. Other “lay-derived” instruments, such as the Older People’s Quality of Life Questionnaire, which was built from the ground up using older people’s own definitions of QoL, are also far superior to the generic HRQoL tools currently in use [38]. In addition, an adapted version of ICECAP-A, namely, ICEpop CAPability measure for older people, has been developed and validated for older adults aged ≥65. This instrument covers the 5 domains of enjoyment, attachment, role, security, and control [29]. Future trials should evaluate the use of appropriate QoL instruments in their study design based on their targeted population.

In addition to these measures of psychological well-being, future trials should consider resilience as a consumer-focused outcome. Resilience may be measured both subjectively through self-reported tools and objectively through acute or longitudinal responses to stressors [39]. Resilience is associated with mental health and well-being and aligns with consumers’ motivations for taking MVM supplements [21,22,40]. Overall, nutritional supplement trials that measure resilience outcomes are still limited and warrant further exploration.

Moving beyond quantitative tools, 1 RCT has compared the qualitative responses of 114 participants enrolled in either MVM or placebo to 3 open-ended questions. These questions asked participants to describe positive, negative, and unusual effects they thought may have occurred from taking the tablets. Thematic analysis revealed the MVM group experienced significantly improved mood (*P* = 0.027) and energy levels (*P* = 0.22), with trends toward improvements in sleep quality [41]. Although perhaps impractical in larger-scale RCT and cohort studies, using qualitative methods for a stratified subgroup of these cohorts may allow for a more nuanced and in-depth investigation of the varied benefits MVMs may provide.

### Linking nutritional adequacy to preservation of QoL

Studies in older adults have shown that adequate nutrition in community-dwelling older adults is associated with better QoL, sleep quality, and higher physical activity levels compared with malnourished individuals [[42], [43], [44]]. However, such a distinction becomes less clear when considering healthy adults who take MVMs to “stay well,” rather than treating a specific deficiency [45]. The use of MVMs to maintain optimal physiological functioning is rarely assessed in trials; however, there are a variety of potential mechanisms underpinning various aspects of preservation of QoL.

First, all B vitamins (excluding folate), vitamin C, iron, and magnesium are required for 1 or multiple stages of intracellular energy production. Consequently, a deficiency in any one of these micronutrients may reduce the rate of energy production and manifest as symptoms of fatigue and reduced vitality [46]. Vitamins C, D, and E play key roles in the protection of tissues from oxidative stress [47]. An adequate intake of vitamin D is associated with a reduction in age-related diseases such as autoimmunity, metabolic disorders, and infections [48]. Furthermore, a variety of micronutrients are involved in cognition; vitamins B-1, B-5, B-9, C, iron, and zinc all have roles in the creation and maintenance of neuronal structures, vitamins B-1, B-5, B-6, B-9, and C are all required for neurotransmitter production, and vitamins B-3, B-5, zinc, magnesium, and iron are important in neurotransmission across synapses [46]. This supports the beneficial effects of MVM supplementation on cognition reported by the COSMOS substudies [49]. Related to this, vitamin E, magnesium, folate, and omega-3 supplementation have been shown to reduce stress and proinflammatory cytokines [50], a potential mechanism behind the lower rates of infections seen in an RCT of MVM supplements [51].

The potential process by which nutritional adequacy may preserve QoL is continuous and multifaceted. Healthy adults may experience transient subclinical insufficiencies due to varying dietary intake, absorption, or utilization [52]. Thus, MVMs may act as a “safety net” to provide background protection against fluctuating insufficiencies. The challenge for future research is to identify those insufficiencies and personalize MVM composition for specific nutritional needs at each point of an individual’s life.

### Recommendations for future study designs

If the hypothesized benefit of MVM supplementation is a subtle improvement in psychosocial well-being, then future trials must be designed accordingly. This would require selecting a population in whom marginal benefit is biologically plausible, using a primary outcome instrument specifically validated for general well-being rather than disease-related disability, measuring outcomes longitudinally, and powering the study to detect small but meaningful changes. For example, a parallel-group, randomized, placebo-controlled trial in middle-aged or older adults with low-normal micronutrient intake, with validated tools such as WHO-5 or PGWBI, and measures of fatigue, vitality, and resilience measured alongside hard clinical endpoints.

Furthermore, because of the subjective nature of QoL outcomes, precautions should be employed to reduce the risk of a placebo effect. This includes a robust double-blinding, allocation concealment, measurement of participant expectations, and longitudinal monitoring of change. Such a design would better test whether MVMs influence vitality, mood, resilience, and subjective well-being, rather than merely disease incidence.

In conclusion, the discrepancy between public behavior and scientific consensus on MVMs is not necessarily a failure of public understanding. It is, in many ways, a misalignment with the scientific inquiry. The public and the scientific community have been asking 2 different questions. Science has been asking, “Does this pill prevent death or disease?” and has concluded “no.” The public has been asking, “Does this pill help me maintain my vitality and feel well?” and, based on their subjective experience, has concluded “yes.”

Until we, the research community, design trials that take the public’s question seriously, we cannot claim to have definitively answered the question. It is entirely possible that MVMs do provide a subtle benefit to psychosocial well-being, a benefit our current clinical trial methodology is conceptually blind to and psychometrically unequipped to see.

## Author contributions

The authors’ responsibilities were as follows – RS: contributed to Writing – original draft, Writing – review & editing, and Project administration; EMC, AG, MW: contributed to Writing – review & editing; and ES, MDAM: contributed to Conceptualization, Supervision, Writing – original draft, and Writing – review & editing.

## Data availability

Data described in the manuscript, code book, and analytic code will not be made available because this is a review of secondary data, and there are no primary data analyzed in this manuscript.

## Funding and Acknowledgement

The authors would like to acknowledge HALEON Singapore for funding the cost of publication for this review.

## Declaration of Generative AI and AI-assisted Technologies in the Writing Process

During the preparation of this work, the authors used Grammarly in order to check for language and grammar accuracy. After using this tool/services, the authors reviewed and edited the content as needed and take full responsibility for the content of the publication.

## Conflict of interest

The authors report no conflicts of interest.

## Figures and Tables

**Table:** 

Search	Search Terms	Hits
#1	“Multivitamin and Mineral” OR “Multivitamin” OR “MVM” OR “Multivitamin and Multimineral” OR “Vitamin and Multimineral” OR “MVMM”	5703
#2	“Quality of Life” OR “QoL” OR “SF-36” OR “SF-12” OR “EQ-5D” OR “EuroQol” OR “EORTC-QLQ” OR “WHOQOL-BREF” OR “HRQoL” OR “Health-Related Quality of Life” OR “Well-being” OR “Life Satisfaction” OR “Self-rated Health” OR “Functional Health”	669,496
#3	#1 AND #2	129
